# The impact of the urban–rural residents’ medical insurance integration on rural residents’ out-of-pocket medical costs: based on the deductible, reimbursement rate, and ceiling line

**DOI:** 10.3389/fpubh.2025.1576978

**Published:** 2025-04-08

**Authors:** Chen Liu, Yue Kong, Qun Su, Huaizhen Xing, Zhongbao Tian

**Affiliations:** College of Economics and Management, Nanjing Agricultural University, Nanjing, China

**Keywords:** URRBMI integration, deductible, reimbursement rate, ceiling line, OOP medical costs

## Abstract

**Background:**

After China implemented the Urban and Rural Residents’ Basic Medical Insurance (URRBMI) integration reform in 2016, medical costs for rural residents remain unalleviated. This might be attributed to the program’s higher deductibles, combined with lower reimbursement rates and ceiling lines.

**Methods:**

Using CHARLS data from 2013 - 2020, this study employs a two-stage Heckman model to examine the impact of changes in deductibles, reimbursement rates, and ceiling lines in the URRBMI reform on out-of-pocket (OOP) medical costs for rural residents. A fixed-effects DID model is also utilized for robustness testing.

**Results:**

(1) Lowering the deductibles for outpatient visits and increasing the outpatient reimbursement rates and ceiling lines can significantly unleash the demand for outpatient visits. The key to reducing residents’ OOP outpatient costs lies in lowering the deductibles and ceiling lines. Meanwhile, the current increase in URRBMI reimbursement levels has not enhanced rural residents’ willingness to seek inpatient visits, and rural residents’ OOP inpatient cost is more sensitive to the inpatient reimbursement rate. (2) Increasing the reimbursement level is conducive to releasing the medical demand of vulnerable groups, such as rural low-income groups and those with poorer health, and is also crucial for reducing the medical burden. The medical behaviours of rural middle-and high-income groups are less influenced by the reimbursement level.

**Conclusion:**

The policy design of medical insurance should give priority to the following: (1) reducing the financial burden of vulnerable groups by lowering deductibles and raising reimbursement ceilings; (2) expanding coverage for major diseases; and (3) expanding the catalogue of reimbursable medicines. These findings offer valuable insights for healthcare reform in developing countries.

## 1 Introduction

Currently, China’s healthcare system faces the challenge of rapidly rising healthcare costs. China’s total healthcare costs and health insurance expenditures are both growing rapidly (1), with an average annual growth rate of 12.2% from 2011 to 2021 (2). In rural China, due to aging and empty—nesting issues, rural middle—aged and older adult residents are more disease—prone (3). At the same time, constrained by disposable income, especially low—income rural residents in China still bear a heavy medical burden and the risk of falling back into poverty due to illness (4). How to reduce the burden of healthcare in rural areas by improving the healthcare insurance system is a topic that needs to be discussed.

**Figure 1:**
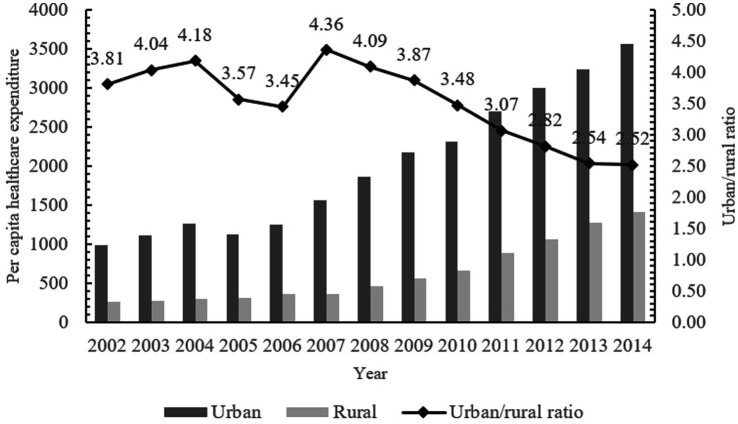
Per capita healthcare expenditure in urban and rural China, 2002–2014.

To improve the utilization of medical resources and reduce the burden of medical care, the Chinese government has successively established three major basic medical insurance systems, including the Urban Employees’ Basic Medical Insurance (UEBMI), the Urban Residents’ Basic Medical Insurance (URBMI) and the New Cooperative Medical Scheme (NCMS). By the end of 2014, the coverage rate of the three basic medical insurance has exceeded 95%, the utilization of medical services by residents has been guaranteed to a certain extent, and the problem of heavy medical burden has been alleviated to a certain extent (5). However, due to the “fragmentation” of the three basic medical insurance systems and significant treatment disparities, the New Rural Cooperative and the Urban Residents’ Medical Insurance differ notably in contribution standards, medicine reimbursement, and designated hospitals (Supplementary Table S1). There are substantial gaps in treatment levels, medical service utilization, and medical burdens between rural and urban residents, as well as between residents and employees (6, 7). Due to insufficient disposable income, the probability of rural residents not seeking medical treatment for a fortnight’s illness is as high as 37.8 percent (8), which inhibits the effective healthcare consumption demand of rural residents, and consequently leads to a large discrepancy in per capita healthcare expenditures between urban and rural areas (Figure 1). To achieve the goal of fair medical protection for urban and rural residents in China, it is urgent to integrate medical insurance between urban and rural areas.

In January 2016, China’s State Council issued the Opinions on Integrating the Basic Medical Insurance System for Urban and Rural Residents, initiating the integration of the urban–rural residents’ medical insurance (URRBMI) system (9).The URRBMI policy requires “six unifications”: unifying medical insurance coverage scope, financing policy, urban—rural protection treatment, the medical insurance catalog, medical institution management, and fund management. After the implementation of the URRBMI Integration policy, URBMMI extended the coverage to all urban and rural residents, expanded the scope of medical services (Supplementary Table S2), and stimulated the demand for medical services among rural residents (5). Studies have shown that the URBMMI policy has reduced the proportion of rural residents who are ‘in need but do not seek medical care’ (10), and the utilization rate of inpatient services has also increased significantly (11).Unified facility management increased the number of medical institutions, enhanced the accessibility of designated hospitals, improved medical service convenience (Supplementary Table S2), and lowered rural residents’ medical care opportunity cost. After the integration, the financing of the former NRCM participants increased by 108.51%, the reimbursement of medicines increased by 163.63%, and the per capita fund expenditure increased by 50.84% (12). It has been shown that China’s URRBMI integration policy plays an important role in promoting health equity (10, 13).

The purpose of the URRBMI policy is to narrow the difference in compensation levels between urban and rural areas, to raise the level of medical resource utilization for rural residents, and to ease the burden of medical care on them. Theoretically, the URRBMI integration policy has raised the level of protection for rural residents by expanding the coverage of medical services and medicines and increasing the number of designated medical institutions, thereby lowering the price of medical services, to reduce the medical costs of rural residents and to promote fairness in the utilization of medical resources between urban and rural areas. However, during the implementation of the URRBMI integration policy, the medical burden of rural residents in China remained high, and a study by Ma et al. showed that the Chinese government’s health and social health expenditures increased year by year, and personal health expenditures also showed high growth from 2003 to 2018 (14). It has also been shown that URRBMI integration cannot share the pressure of medical care for rural high-disease-burden groups (15, 16). The failure of the URRBMI integration to reduce medical costs for rural residents may be attributable to structural flaws in the current compensation mechanism—a core mechanism of health insurance systems whose design directly determines the capacity to mitigate individual medical burdens.

The compensation program is an important part of the URRBMI policy. China’s medical insurance compensation program standards have three main aspects. First is the deductible. After incurring medical costs, a participant pays out-of-pocket for a certain amount, and only costs above this amount are covered by the social insurance operator. Second is the reimbursement rate, where the participant and the social insurance operator share medical costs at certain percentages. Third is the ceiling line. Once the social insurance operator has paid up to a prescribed amount for a participant’s medical costs, no further payment is made. In 2016, the Chinese government proposed in the specific requirements for the harmonization of the URRBMI protection benefits that it should ‘further improve outpatient integration, and gradually provide the level of outpatient protection’, and required that the reimbursement ratio for inpatient hospitalization be maintained at around 75%. After 2016, most cities initiated integration. Provincial human resources and social security departments issued URRBMI integration implementation plans, setting basic requirements for funding, outpatient, and inpatient treatment. Municipalities in each province then promoted reform according to local conditions, announcing deductible, reimbursement rates, and ceiling lines, resulting in significant differences in specific compensation levels among provinces. Most cities have implemented a tiered compensation policy, to encourage residents to seek treatment at primary hospitals and improve the efficiency of medical resource utilization. Within the scope of the policy, different deductible and reimbursement rates are set for first-, second-, and third-tier hospitals, with the deductible and the reimbursement rates decreasing as the level of the hospital rises. Although the URRBMI integration policy has played a positive role in adjusting the level of compensation, the following problems exist in practice: from the viewpoint of policy documents around the world, the level of URRBMI compensation has been rising slowly. The current URRBMI integration policy has not achieved the optimal reimbursement rates (around 70%) (17). The burden of residents’ financing and contributions has been increasing year by year (18), and there are even withdrawals and abandonment of insurance, and the actual medical burden and cata-strophic expenditures of rural residents have not been reduced (19). Therefore, the fact that URRBMI integration has not reduced the medical costs of rural residents may be related to the current low level of URRBMI compensation in China.

There has been a rich discussion of research on the impact of medical insurance compensation programs on medical costs. Studies have shown that the deductible, the reimbursement rate, and the ceiling line are closely related to the medical costs of the population. Concerning the deductible, a study of commercial insurance in the United States found that companies that replaced their employees’ commercial insurance with medical insurance with a high deductible experienced a significant decrease in employee medical costs (20). A similar effect was verified in studies on purchased drug programs for older adults care in the US, which showed that the setting of a deductible significantly reduced medical costs for the older adults (21–23). A study by Remmerswaal et al. on the two types of universal medical insurance in the Netherlands suggests that healthcare programs with lower deductible policies are more able to save on medical costs (24) and that consumers’ expectations for higher expectation that medical costs will reach the deductible, the more medical consumers will consume (21). In terms of reimbursement rates, a study by Shen et al. on medical insurance policies in a Chinese city found that increasing the reimbursement rate of medical insurance led to an increase in the utilization of primary outpatient services and a significant decrease in the utilization of other healthcare facilities (25). A study by Chinese scholar Zhu Fengmei on the ceiling line of employee health insurance showed that the cancelation of the outpatient ceiling line did not significantly promote outpatient utilization and outpatient costs and that the setting of the ceiling line could alleviate the expenditure of the inpatient medical insurance fund to a certain extent (26). However, some scholars based on the study of urban and rural residents’ major illness medical insurance in Shanghai showed that the effect of setting the ceiling line for major illness medical insurance on the balance of income and expenditure of the medical insurance fund was not significant (27). Some scholars have discussed the substitution effect of the outpatient ceiling on the use of inpatient medical resources, and the study shows that the cancelation of the outpatient ceiling can save inpatient medical insurance expenditure, which can simultaneously achieve the win-win effect of improving the treatment and saving the medical insurance fund (28).Reviewing the above studies, the impact of medical insurance on participants’ medical resource use and costs is closely related to the setting of the deductible, reimbursement rate, and ceiling line. However, no study has incorporated the compensation level into the framework of URRBMI’s impact on rural residents’ medical costs. Therefore, it is crucial to include a discussion of the level of coverage in the exploration of the impact of URRBMI integration on residents’ medical costs.

Combining the above—mentioned realistic background and literature, it can be deduced that even after the implementation of the URRBMI integration policy, rural residents’ medical costs in China remain high. There is a large gap between different rural income groups, likely related to differences in local compensation levels. Behind this may be issues such as a too—high deductible, low reimbursement for actual medical costs, or low reimbursement ratios and ceiling lines. Problems. China’s URRBMI integration policy has completed the transition from ‘without’ to ‘with’, and the focus of the current policy research should be on how to further set the level of treatment, to better enhance the utilization of healthcare resources for rural residents, narrow the gap within the countryside, and further reduce the medical burden among different groups. Therefore, it is necessary to improve the assessment framework between the existing URRBMI integration policy and the medical costs of rural residents, based on which to explore the mitigation options for the increased medical burden of rural residents from the perspective of the level of protection. In this study, using the four periods of data from the China Health and Retirement Longitudinal Study (CHARLS) in 2013, 2015, 2018, and 2020, and based on the tracking research before and after the pilot integration by CHARLS, the Heckman two-stage regression method and double difference method to analyze the impacts of protection level and financing level settings on OOP medical costs of Chinese rural residents in each region, respectively. There are two main contributions: (1) In terms of research perspective, this study has improved the analytical framework of the impact of China’s URRBMI integration reform on the medical costs of rural residents from the perspectives of the deductible, the reimbursement ratio, and the ceiling line. (2) In terms of data processing, this paper combed the data on the treatment level of the URRBMI integration in 125 cities in China, which is of positive significance for better improving the content of the URRBMI integration policy.

## 2 Theoretical analysis and research hypotheses

Consider the impact of changes in compensation levels on OOP medical costs of rural residents after the implementation of the URRBMI integration policy. Assuming that the prevalence rate of rural residents is *α* (αϵ[0,1]) and that their utility is the sum of their expected utility when healthy and their expected utility when sick, the optimal amount of individual consumption of healthcare services utilized, $Q_{m}^{*}$ is used to maximize the utility of healthcare services utilization:


(1) $$\max\;U=\alpha \times U_{uh}+\left(1-\alpha \right)\times U_{h}$$

$$s.t\;Q_{cuh}+P\times Q_{m}=y$$

$$Q_{cuh}\geq 0$$

$$Q_{m}\geq 0$$

The specific functional form of $Q_{m}^{*}$ is: $\partial U/\partial Q_{m}^{*}=-\alpha \times P+\alpha \times v^{\prime }\left(Q_{m}^{*}\right)=0$ , $v^{\prime }\left(Q_{m}^{*}\right)=P$ , *P* is the price of healthcare service utilization, and $Q_{m}^{*}=H\left(P\right)$ . The utility function satisfies $v^{\prime }\left(.\right)>0,v^{\prime \prime }\left(.\right)<0$ when the medical service is a necessity. A decrease in price triggers an increase in consumption. At this point, $z=H\left(P\right)\times P$ is the medical consumption of the enrollee. Rural residents’ access to health care is constrained by their disposable income, if rural residents’ disposable income is most sufficient to cover health care consumption, i.e., y≤z . If the income level of rural residents cannot afford the optimal consumption of health care services, only $Q_{m}^{*}=y/P$ can be achieved.

Rural residents at different income levels may have different healthcare behaviors and healthcare burdens when faced with the same level of disease and probability of illness, due to their different economic levels. Residents are categorized into low-income and high-income levels according to their income levels. Medical insurance for urban and rural residents takes the form of charging a fixed premium, with a premium level of *t* and a coverage level of *b*.

The URRBMI integration policy has changed the prices of medical services faced by participants and brought about changes in the amount of demand for medical care by participants. Although urban and rural residents’ health insurance gives financing subsidies to some groups, it does not directly change the income constraints of residents. Let the optimal utilization of medical resources for low-income residents after the co-ordination of urban and rural residents’ health insurance be $Q_{\mathrm{ml}}^{*}$ , $Q_{\mathrm{ml}}^{*}=\left[z-t\right]/\left(1-b\right)P$ . At that time when t<b *z*, $Q_{\mathrm{ml}}^{*}>Q_{m}^{*}$ . That is, when the individual contribution level of the rural low-income group is lower than the guaranteed level, the medical service utilization increases. The consumption of medical services of high-income residents is $Q_{mh}^{*}=H\left[\left(1-b\right)P\right]$ , which satisfies ${v^{\prime }}^{\prime }\left(.\right)<0,H^{\prime }\left(.\right)<0$ . Since $\left(1-b\right)P<P$ , it can be seen that $Q_{mh}^{*}>Q_{m}^{*}$ . The utilization of healthcare resources by high-income rural residents also rises after the integration of urban and rural residents’ healthcare insurance.

When the level of URRBMI coverage is increased, the changes in the most healthcare consumption of low-income rural residents and high-income rural residents are $\partial Q_{\mathrm{ml}}^{*}/\partial b=\left(z-b\right)/{\left(1-b\right)}^{2}P>0$ ; $\partial Q_{mh}^{*}/\partial b=-H^{\prime }\left[\left(1-b\right)P\right]>0$ . When the level of entitlement is increased, the effect of the URRBMI co-ordination on the healthcare resource utilization for both low-income rural residents and high-income rural residents is boosted, but the exact amount of increase is related to the form of the price function.

$H\left(\text{.}\right)$ . In addition, when the level of protection is raised, the medical costs of residents are subsequently increased.

Low-income participants are affected by income constraints and their medical needs are not fully realized. If the medical needs of low-income rural residents have been fully released and not fulfilled, $Y_{opl}=z-t$ is the maximum amount of medical expenditure available to this group, even if increasing the level of protection does not affect OOP medical expenses $Y_{opl}$ However, at present, the health awareness of low-income rural groups in China is relatively weak, and the medical needs of rural residents may not be fully released, so raising the level of protection is still of some significance to the medical care and medical burden of low-income rural groups. For the high-income rural group, the OOP medical costs are $Y_{oph}=H\left[\left(1-b\right)P\right]\left(1-b\right)P$ , and with the increase of the protection level *b*, $\frac{\partial Y_{oph}}{\partial b}=-H^{\prime }\left({\left[\left(1-b\right)P\right]}_{1}-b\right)P^{2}-H\left[\left(1-b\right)P\right]P$ . From this, we can see that the OOP medical costs of the high-income rural residents are ssubjected tothe opposite influences of their demand and price. The exact direction and magnitude of change are also related to the specific form of $H\left(\text{.}\right)$ .

The URRBMI integration policy of raising the level of compensation aims to release the medical demand of rural residents, increase the utilization rate of medical resources, and reduce the medical costs of rural residents. Derived from the theoretical model of individual utility maximization, it can be seen that raising the level of compensation (including lowering the deductible, increasing the reimbursement rates, and the ceiling line) lowers the price of medical services and reduces medical costs. Specifically, the lower the deductible in the compensation level of the URRBMI integration policy, the more it can promote the amount of medical resource service utilization by insured residents for minor illnesses, and at the same time, the price effect triggers rural residents to reduce their OOP expenses and their medical burden is alleviated; when the reimbursement rates are increased, the price of medical service utilization faced by rural residents also decreases, and the rural residents’ OOP medical costs are reduced, and their medical costs is alleviated; The higher the ceiling line, the more the OOP costs of hospitalization for major illnesses can be reduced, both reducing the medical burden; the higher the financing subsidy, the higher the actual level of financing enjoyed by the residents, and the burden of contribution is reduced. Distinguish between income levels, if the medical needs of low-income rural residents have been fully released, raising the level of protection to ease their medical burden does not have an impact, but at present, China’s residents are still under-utilized in terms of medical resources, and low-income rural residents are more sensitive to the price of medical services, so raising the level of treatment is still of great significance to the low-income groups in rural areas in terms of releasing their medical needs and easing their costs. For high-income rural residents, whose demand for medical care is less constrained by their income, there is also the possibility of releasing demand for medical care while reducing OOP medical costs.

Hypothesis H1 is proposed based on the above analysis:

*H*1: Increasing the level of compensation for URRBMI, including lowering the deductible and increasing the reimbursement rate and ceiling line, can reduce the price of medical services for rural residents, which will ultimately help to alleviate the OOP medical costs of rural residents.

## 3 Data sources and research methodology

### 3.1 Data

Data were obtained from the China Health and Retirement Longitudinal Study (CHARLS), which has conducted five rounds of investigations. This study utilized data in 2013, 2015, 2018, and 2020. Data for 2011 was not used because only a few provinces implemented reforms to integrate URRBMI in 2011. The CHARLS sample is broadly representative, and the data contain detailed information on individual characteristics, healthcare behaviors, and healthcare costs, which fits the theme of this study. In addition, the sample has a low rate of lost visits (29–31).

This study uses data from the CHARLS database for the years 2013, 2015, 2018, and 2020, and data from 2011 were not used, mainly because, in 2011, only a very few provinces implemented the policy of Integration health insurance for urban and rural residents, and the sample was too small, so the sample from this year was excluded from this paper.

In addition to data on individual characteristics of residents and their access to healthcare, data on treatment levels in this study were compiled from the official government websites of the 125 cities in the CHARLS sample, mainly the ‘official websites of the municipal people’s government’ or ‘official websites of the Bureau of Human Resources and Social Security’ of each city in China’. In this paper, we collate policy documents related to URRBMI integration from the official government websites of each city from 2015 to 2020 and sort out the specific timing of URRBMI integration in each city (Supplementary Table S3), as well as the content of specific outpatient and inpatient compensation programs (including the deductible, reimbursement rate and ceiling line for outpatient and inpatient medical institutions at all levels). To ensure the integrity of the data, for the data on treatment levels that were unavailable through inquiry, this study filled such missing values with the data of the same city from the previous year.

### 3.2 Variable selection and data description

The dependent variables in this study include medical resource utilization and OOP medical costs. Among them, medical resource utilization includes outpatient visits and inpatient visits, which are measured by the CHARLS questionnaire ‘whether outpatient visit in the last month’ and ‘whether inpatient stays in the last year’, respectively. The OOP medical costs variable includes outpatient OOP costs and inpatient OOP costs. Outpatient OOP costs measure the actual outpatient costs paid by the respondent in the most recent month after deducting reimbursements, and inpatient OOP costs measure the actual inpatient costs paid by the respondent in the most recent year after deducting reimbursements.

The core dependent variables in this study are the URRBMI treatment levels, including the deductible, reimbursement rates, and the ceiling line.

The deductible, also known as the threshold fee, is one of the compensation rules of medical insurance. The deductible in URRBMI refers to the medical costs that the insured person has to bear on his own before enjoying the reimbursement of URRBMI, and after exceeding the deductible, the rest of the medical costs will be reimbursed by the reimbursement standard of each place. The aim is to mitigate the problem of moral hazard and control medical costs. The setting of deductibles can reduce unnecessary medical services to a certain extent (32), so the reasonable setting of the starting line is of great significance in alleviating the problem of moral hazard and controlling medical costs. In the process of URRBMI integration, different deductibles are set in different places according to different levels of hospitals. Most provinces and cities in China do not set outpatient deductibles for URRBMI integration, meaning that all outpatient costs below the ceiling line for residents are reimbursed on a pro-rata basis. Some provinces and cities, such as Zhangzhou City in Fujian Province, Taizhou City in Jiangsu Province, and Yancheng City in Jiangsu Province, set a deductible of RMB 10–50, while Beijing City, Shijiazhuang City in Hebei Province, Yangzhou City in Jiangsu Province, Jiamusi City in Heilongjiang Province, Jinzhou City in Liaoning Province, Tianjin City, and Lishui City in Zhejiang Province set a deductible of RMB 100–500. In terms of hospitalization deductibles, URRBMI has different deductibles for designated medical institutions at different levels. Depending on the level of the hospital, the hospitalization deductible increases in turn. Generally speaking, the hospitalization deductible for different levels of hospitals shows a gradient, with first-level medical institutions and community health service institutions having the lowest deductible, second-level medical institutions having slightly higher deductibles and third-level medical institutions having the highest deductible.

The reimbursement rates are the proportion of expenses paid by the medical insurance fund above the deductible and below the ceiling line. Currently, outpatient reimbursement rates in most of China’s URRBMI integration areas range from 50 to 75% (Supplementary Table S4). The reimbursement rate for first-level hospitalization in most integrated cities ranges from 65 to 85%, second-level hospitalization from 60 to 80%, and third-level hospitalization from 50 to 70%, with the reimbursement rate decreasing in a graded manner (Supplementary Table S5). Higher reimbursement rates for primary hospitals can effectively guide patients to primary hospitals (33) and alleviate the problem of strained utilization of healthcare resources. However, the current differential reimbursement policy in the URRBMI integration policy has limited effect (34).

The URRBMI ceiling or maximum payment limit is the maximum amount of compensation from the medical insurance fund that a participant can receive within 1 year, to reasonably control the costs paid by the medical insurance fund. The vast majority of URRBMI-integrated cities in China have set outpatient ceiling lines ranging from RMB 50 to RMB 4,000. A few areas, such as Ji’an City and Ganzhou City in Jiangxi Province, Foshan City and Qingyuan City in Guangdong Province, Shanghai City and Hangzhou City in Zhejiang Province, do not have an outpatient ceiling. Studies have shown that the cancelation of the outpatient ceiling line can achieve savings in inpatient health insurance costs, thus realizing the win-win effect of saving funds and improving treatment (28). For the inpatient ceiling line, each integrated city sets different amounts of inpatient ceiling lines according to the local economic development situation. There are large differences in the setting of the ceiling line across the region, such as the hospitalization ceiling line of RMB 40,000 in 2018 for Chuxiongzhou Yi Autonomous Region in Yunnan Province and Lanzhou City in Gansu Province, and RMB 50,000 in 2018 and 2020 for Zhangye City in Gansu Province (Supplementary Table S6).

In addition, it should be noted that most of the cities in China that have achieved URRBMI integration do not have a hierarchical setup of reimbursement for the level of medical institutions in the outpatient reimbursement, while for the inpatient reimbursement, all the cities that have achieved integration have a hierarchical setup of reimbursement, i.e., they differentiate between first-level, second-level, and third-level medical institutions in setting up their reimbursement programs, with first-level medical institutions having the lowest deductible and the highest reimbursement rates, while third-level medical institutions have the highest deductible and the lowest reimbursement rates, and second-level medical institutions having reimbursement levels in between. The deductible is lowest and the reimbursement rate is highest in first-level medical institutions, the deductible is highest and the reimbursement rate is lowest in third-level medical institutions, and the level of reimbursement in second-level medical institutions is in between. To avoid the problem of covariance that might be caused by putting the reimbursement levels of all levels of hospitals into the same equation, this study chooses the outpatient and inpatient reimbursement levels of Level 1 medical institutions as the core independent variables to measure the reimbursement levels.

In terms of the setting of control variables, Grossman’s health needs model proposes that reasons such as age, income, education, uncertainty of disease, and price of healthcare services affect individual health needs (35). Referring to the study of Zhu and Wang, when exploring the impact of URRBMI integration on residents’ medical costs, residents’ demographic characteristics, health variables, health risk awareness, and household income variables should be included (36). Combined with the information from the CHARLS database, individual characteristics in this study include age, gender, and marriage, health features involving health status and disability status, health awareness comprises smoking, drinking, regular check-ups, and physical exercise, income characteristics represent the participant’s total income in the past year, combining annual wage, self-employment income, pension income, agricultural income, and personal business income. The descriptive statistics of this study are shown in Table 1. The meanings of the variables are shown in Supplementary Table S7.

**Table 1:** Results of descriptive statistics of variables.

Variables	2013 (*N* = 11,154)	2015 (*N* = 11,552)	2018 (*N* = 13,837)	2020 (*N* = 14,816)
Age (Mean, SD)	61.73 (9.60)	60.70 (10.07)	62.84 (9.89)	61.75(9.87)
Sex				
Female	5.958 (53.4%)	6.006 (52.0%)	7.461 (53.9%)	7.691 (54.8%)
Male	5.196 (46.6%)	5.546 (48.0%)	6.376 (46.1%)	6.354 (45.2%)
Education level				
High school and above	669(6.0%)	7.162(6.2%)	844(6%)	963(6.5%)
Marriage				
Unmarried	1.492 (13.4%)	1.402 (12.1%)	2.044 (14.8%)	7.691 (54.8%)
Married	9.662 (86.6%)	10.150 (87.9%)	11.793 (85.2%)	6.354 (45.2%)
Health status				
Very Unhealthy	363 (6.5%)	383 (6.9%)	814 (6.3%)	1.547 (12.1%)
Quite unhealthy	1.412 (25.3%)	1.169 (21.0%)	2.885 (22.3%)	1.480 (11.6%)
Fairly healthy	2.682 (48.1%)	2.779 (49.9%)	6.253 (48.4%)	6.312 (49.3%)
Fairly healthy	700 (12.6%)	591 (10.6%)	1.447 (11.2%)	2.499 (19.5%)
Very healthy	417 (7.5%)	643 (11.6%)	1.533 (11.9%)	960 (7.5%)
Disabled or not				
No	9.839 (88.2%)	10.190 (88.2%)	12.639 (91.3%)	13.298(89.8%)
Yes	1.315 (11.8%)	1.362 (11.8%)	1.198 (8.7%)	1.518(10.2%)
Physical activity				
No	830 (22.0%)	2.401 (43.0%)	2.562 (18.5%)	8.375 (59.7%)
Yes	2.939 (78.0%)	3.189 (57.0%)	11.259 (81.5%)	5.646 (40.3%)
Smoking				
No	750 (32.9%)	6.409 (65.5%)	13.387 (98.4%)	6.327 (96.1%)
Yes	1.531 (67.1%)	3.375 (34.5%)	214 (1.6%)	260 (3.9%)
Drinking				
No	8.261 (74.3%)	8.386 (72.7%)	10.265 (74.3%)	9.305 (66.4%)
Yes	2.855 (25.7%)	3.146 (27.3%)	3.555 (25.7%)	4.714 (33.6%)
Routine physical examination				
No	9.012 (80.8%)	8.938 (77.4%)	9.907 (71.6%)	7.849 (56.3%)
Yes	2.142 (19.2%)	2.614 (22.6%)	3.930 (28.4%)	6.102 (43.7%)
Outpatient visits				
No	8.635 (77.7%)	9.188 (79.6%)	11.586 (83.8%)	11.152 (79.5%)
Yes	2.474 (22.3%)	2.352 (20.4%)	2.248 (16.2%)	2.877 (20.5%)
Inpatient visits				
No	9.668 (86.8%)	10.018 (86.8%)	11.486 (83.0%)	14.385 (97.1%)
Yes	1.470 (13.2%)	1.525 (13.2%)	2.348 (17.0%)	431 (2.9%)
OOP outpatient costs (Mean, SD)	766.96 (2719.10)	929.61 (2728.86)	1148.72 (4750.22)	1154.26 (3028.44)
OOP hospitalization costs (Mean, SD)	8,085.72 (13,177.42)	9,700.50 (17,152.37)	13,109.30 (23,122.48)	13,828.13 (19,235.45)
Total household income (Mean, SD)	19,034.20 (49,663.53)	13,032.76 (23,784.60)	19,649.77 (71,544.27)	35,836.31 (28,642.84)

Mean (SD) was conducted for continuous variables; n (%) and chi-square test were conducted for categorical variables.

**Figure 2:**
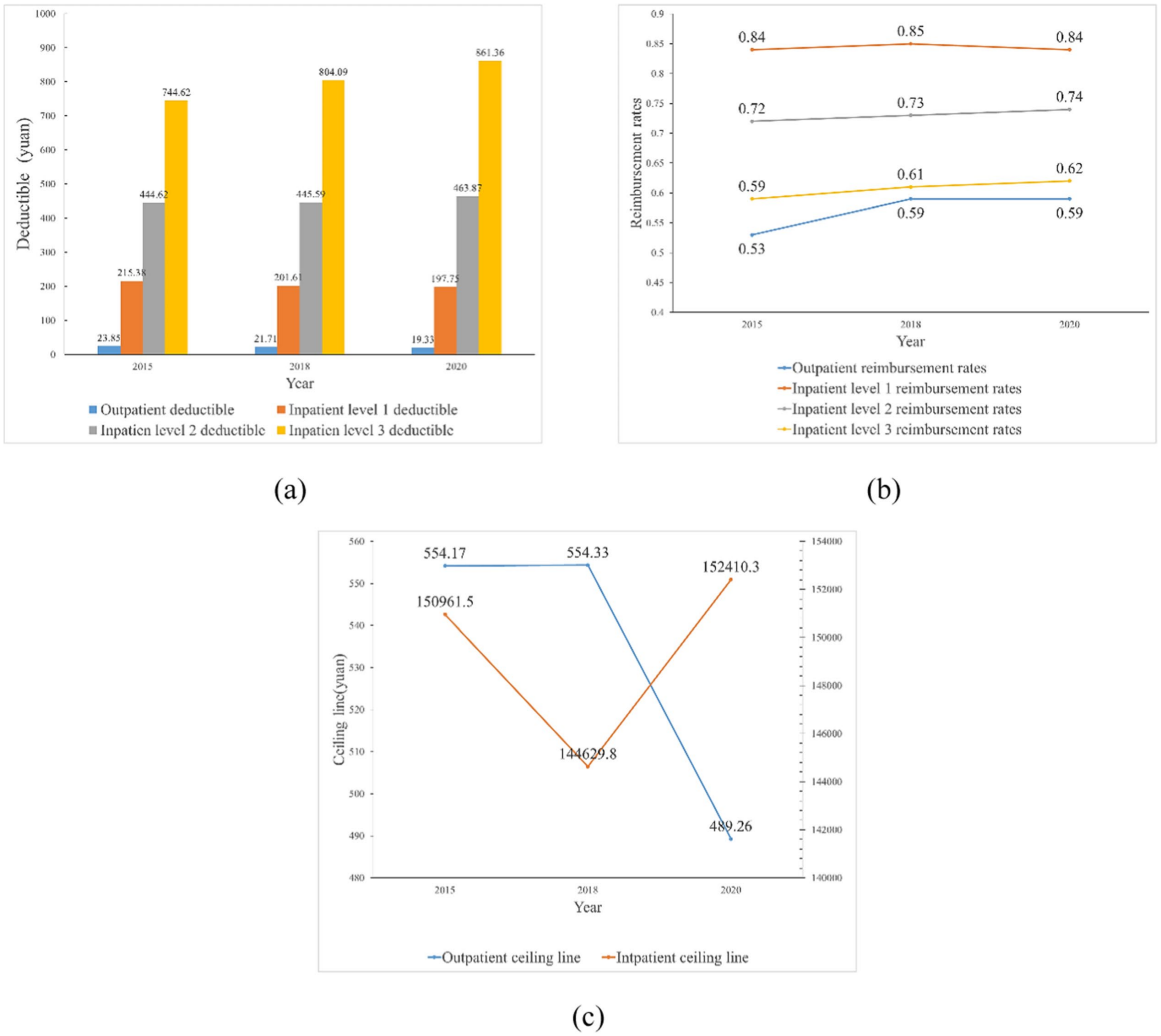
Trends in outpatient and inpatient deductible, reimbursement rates, and ceiling lines, 2015–2020 (mean) **(a)** Trends in outpatient and inpatient deductible, 2015–2020 (mean); **(b)** Trends in outpatient and inpatient reimbursement rates, 2015–2020 (mean); **(c)** Trends in outpatient and inpatient ceiling lines, 2015–2020 (mean). The data in figures **(a–c)** are sourced from the sample data of this study.

Figure 2 further illustrates the results of China’s URRBMI Integration policy in terms of compensation levels over time. The results in Figure 2 show that from 2015 to 2020, in the outpatient compensation level of China’s URRBMI, the outpatient deductible decreased and the outpatient reimbursement rates tended to increase, while the outpatient ceiling line did not change much, or even decrease. The possible reason for this is that some cities only opened the implementation program of URRBMI integration in 2020 and set a lower level of outpatient ceiling line at the early stage of policy implementation, thus the overall average value has decreased. In the level of inpatient reimbursement, the deductible for primary medical facilities declines by about $18 between 2015 and 2020, the deductible for secondary healthcare facilities rises slightly, and the deductible for tertiary healthcare facilities rises sharply. This is closely related to China’s measures to encourage hierarchical diagnosis and treatment and to encourage residents to visit primary care institutions. However, the change in hospitalization reimbursement rates is insignificant, with a negligible increase in 2018 and 2020 compared to 2015. In addition, there was a small increase in the amount of the inpatient ceiling line from 2015 to 2020. Overall, after the URRBMI integration policy was fully rolled out nationwide in 2016, the overall level of compensation for URRBMI has risen, but the rate of improvement has been slow.

### 3.3 Modeling approach

This study focuses on the impact of the compensation level of the URRBMI integration on the medical costs of rural residents. Firstly, this study analyses the impact of the compensation level of URRBMI on rural residents’ OOP medical costs by constructing the Heckman two-stage method. Second, it explores the heterogeneous impact of the compensation level of the URRBMI integration on rural residents with different incomes. In the robustness test section, to further validate the validity of the conclusions, this study utilized the difference-in-differences approach for verification.

#### 3.3.1 Heckman two-stage model

Since the healthcare behavior of individual residents is influenced by factors such as income constraints, i.e., ability to pay, health awareness, and accessibility to healthcare, there is a phenomenon that some groups of people do not seek healthcare when they are ill. To avoid the problem of selectivity bias in the sample, the two-stage model proposed by Heckman (37) was used in this study, see Equation 2:


(2) $${P_{i}}^{*}=\delta_{0}+\delta_{1}X_{i}+\varepsilon_{i}$$

where ${P_{i}}^{*}$ is an unobservable latent variable, if ${P_{i}}^{*}>0$ , then $P_{i}=1$ ; if ${P_{i}}^{*}\leq 0$ ^* ≤ 0, then $P_{i}=0$ .

Set the Probit model for the first stage, see Equation 3:


(3) $$\mathrm{Prob}\left(P_{i}=1|X_{i}\right)=\emptyset \left(\delta_{0}+\delta_{1}X_{i}\right)$$

$P_{i}$ is the resident’s choice of consultation after illness, in the consultation choice model, if $P_{i}=1$ , it means that the resident receives outpatient treatment or inpatient treatment after illness. $X_{i}$ is the factors affecting the resident’s choice of consultation, which mainly includes the treatment level variables and the control variables resident’s characteristics such as age, gender, marital status and education, the self-assessment of health status, whether disability and other health status variables, whether to drink alcohol or not, smoking, health awareness variables such as whether to participate in routine medical check-ups and physical exercise, and personal annual income variables, $\delta_{1}$ is the coefficient of the above dependent variables. $\delta_{0}$ is a constant term and $\varepsilon_{i}$ is a random error term.

The inverse Mills ratio was further calculated for each observation using the formula, see Equation 4:


(4) $$\lambda_{i}=\frac{\emptyset \left({\hat{\delta }}_{0}+{\hat{\delta }}_{1}X_{i}\right)}{\varphi \left({\hat{\delta }}_{0}+{\hat{\delta }}_{1}X_{i}\right)}$$

where $\emptyset \left({\hat{\delta }}_{0}+{\hat{\delta }}_{1}X_{i}\right)$ represents the density function under the ard normal distribution and $\varphi \left({\hat{\delta }}_{0}+{\hat{\delta }}_{1}X_{i}\right)$ represents the cumulative probability distribution function.

Including the inverse Mills ratio from the first stage as a control variable in the regression equation yields the second stage of the analytical equation affecting the medical costs, see Equation 5:


(5) $$y_{i}=\alpha_{1}X_{i}+\alpha_{2}\lambda_{i}$$

Where $y_{i}$ represents the correlation variables of healthcare resource utilization and healthcare costs, $\lambda_{i}$ is the inverse Mills ratio calculated according to Equation 4, and if the coefficient $\alpha_{2}$ is significantly not equal to 0, then Heckman’s method should be used to deal with the sample selection bias problem.

#### 3.3.2 Difference in difference model

This study employed the Difference in difference model (DID) model to analyze China’s URRBMI policy impact on residents’ healthcare resource utilization and medical costs. The DID model was first applied to the field of economics by Ashenfelter (38), and Chinese scholars Zhou and Chen (39) introduced the DID model to China and applied it to the evaluation of public policies. The DID model estimates the average treatment effect of a policy or intervention by comparing the difference between the treatment and control groups at two points in time before and after the policy or intervention. Assessing the policy effect under the traditional method is mainly done by setting a dummy variable for whether the policy occurs or not and then regressing it, in contrast, the model setup of the DID method is more scientific and can estimate the policy effect more accurately, and it can also avoid the problem of endogeneity to a certain extent. The nationwide implementation of the URRBMI integrated policy in 2016 served as a quasi-natural experiment. The study considered 2016 as the policy implementation year, using data from 2013 and 2015 as pre-policy samples and data from 2018 and 2020 as post-policy samples. Residents in URRBMI or NCMS in 2013 and 2015 who continued with URRBMI in 2018 and 2020 formed the experimental group, while those in URBMI or NCMS in 2013 or 2015, not join URRBMI during the study period, comprised the control group.

This paper further controls for time and area factors in the DID model. Referring to the research of He and Shen, Shen, Chen and Jin (40–42), region-fixed effects were included to control for region characteristics that did not change over time. As CHARLS is a long-term tracking data, this study also draws on Rubin and uses the time-fixed effects model (43), which has the advantage of excluding the effect of unobservable factors that are constant over time and controlling for changes in the year of URBMMI implementation. Gardiner and Luo point out that fixed-effects models control for individual trends over time and are more realistic, whereas random-effects models while controlling for variation between data, do not identify all differences between individuals and therefore underestimate standard errors (44). Therefore, this study adopted a fixed-effects double-difference model in the assessment concerning the integration of urban and rural residents’ medical insurance and residents’ medical costs.

Drawing on model set by Li et al. and Huang and Wu (45, 46), the model is set as follows, see Equations 6, 7:


(6) $$U_{it}=\beta_{0}+\beta_{1}DID_{\mathrm{it}}+\beta_{2}\mathrm{Treatmen}t_{i}+\beta_{3}Post_{t}+\sum_{m}\alpha_{m}X_{\mathrm{it}}^{m}+\mu_{\mathrm{it}}$$

(7) $$DID_{\mathrm{it}}=\mathrm{Treatmen}t_{i}\times Post_{t}$$

The dependent variables and control variables are the same as those in the previous section. To further examine the impact of the level of coverage in the URRBMI Integration on the utilization of medical resources and the medical costs on the residents, this study further distinguishes the samples into the ‘with a deductible’ and ‘without a deductible’ groups according to the level of reimbursement of the integration city, ‘high reimbursement rate’ and “low reimbursement rate” groups, and “with ceiling line” and “without ceiling line” groups are discussed separately. The median is used as the basis for the classification of reimbursement rates.

## 4 Results and analysis

### 4.1 Baseline regression

To explore the impact of changes in the compensation level of URRBMI integration on the use of healthcare resources and OOP medical costs of rural residents, this part uses data from the CHARLS database for 2013, 2015, 2018, and 2020, as well as collating data on the compensation level of URRBMI integration in 2015, 2018, and 2020 in each integrated city, and adopts a Heckman two-stage regression to conduct and explore, and the results obtained are shown in Table 2.

**Table 2:** Heckman’s two-stage regression results.

Variables	Outpatient visit model		Inpatient visit model	
	Stage 1: whether outpatient	Stage 2: OOP costs for outpatient visits	Stage 1: whether inpatient	Stage 2: OOP costs for inpatient visits
Outpatient deductible	−0.002^**^	0.569^***^		
	(0.001)	(0.155)		
Outpatient reimbursement rate	0.646^***^	−0.075		
	(0.210)	(0.884)		
Outpatient ceiling line	0.025^***^	−0.139^**^		
	(0.007)	(0.067)		
Inpatient			0.007	0.300
			(0.062)	(0.274)
Deductible			0.239	−3.072^**^
			(0.156)	(1.295)
Inpatient reimbursement rate			−0.002	−0.061
			(0.022)	(0.086)
Control variables	YES	YES	YES	YES
Inverse mills ratio		−1.351		1.670^**^
		(1.190)		(0.708)
Constant term	−0.588^***^	7.992^***^	−0.761	6.277^***^
	(0.212)	(1.623)	0.453	2.147
N	9,741	67	10,205	439

Robust standard errors in parentheses, ^***^, ^**^, and ^*^ are significance levels of 1, 5, and 10%, respectively.

The regression results of the Heckman two-stage model show that the inverse Mills ratio of the outpatient model is not significant for outpatient OOP costs, indicating that there is no sample selection bias problem in the sample’s outpatient visit behavior, while in the inpatient model, the coefficient of the inverse Mills ratio is significant, and there is a sample selection bias problem, which is solved by the Heckman two-stage model. In the outpatient visit model, the estimation results of the first stage: whether or not to visit the outpatient clinic after illness show that reducing the outpatient deductible, increasing the outpatient reimbursement ratio, and raising the outpatient ceiling line significantly increase the probability of rural residents’ outpatient visit, and the demand for outpatient visit is released after raising the treatment level. Stage 2: The estimation results of the outpatient OOP costs model show that controlling for other factors remaining unchanged, a 1% increase in the outpatient deductible increases rural residents’ outpatient OOP costs by 0.569%; a 1% increase in the outpatient ceiling line reduces residents’ outpatient OOP costs by 0.139%, which also suggests that residents of the current URRBMI integrated area have outpatient consultation costs that exceed the established outpatient ceiling line, and that raising the outpatient ceiling line will, to a certain extent, alleviate the problem of high outpatient OOP expenses for rural residents. At the same time, the current increase in the outpatient reimbursement rate for URRBMI has not effectively alleviated the problem of high OOP costs for rural residents, possibly because (1) the current reimbursement rate is growing slowly; (2) the medical insurance catalog has not been effectively expanded, and the types of illnesses of residents are not being reimbursed. This has led to an increase in the nominal reimbursement rate, while the actual reimbursement rate is low.

In the inpatient visit model, the estimation results of the first stage: whether or not to be inpatient after illness show that the current compensation level enhancement of the URRBMI integration does not effectively enhance the willingness of insured rural residents to be inpatient, i.e., the elasticity of demand of the compensation level on the willingness of rural residents to be inpatient is relatively small. The estimation results of the second stage: the inpatient OOP costs model show that the reimbursement rate increase in the URRBMI compensation level can significantly reduce the inpatient OOP costs of rural residents, while the deductible and ceiling line do not play a significant role in the OOP costs of inpatient of rural residents.

In conclusion, for outpatient visits, the outpatient deductible, reimbursement rate, and ceiling line are all determinants of rural residents’ outpatient visits to hospitals, and the threshold amounts of the outpatient deductible and ceiling line are crucial to residents’ outpatient costs. For inpatient visits, the compensation level is not a major factor in rural residents’ decision to seek outpatient visits; the price elasticity of demand for inpatient visits is lower than that for outpatient visits, but an increase in the reimbursement rate can also effectively alleviate the problem of high inpatient costs.

### 4.2 Heterogeneity analysis

Table 2 discusses the impact of URRBMI compensation levels on rural residents’ outpatient and inpatient visit behaviors and outpatient and inpatient OOP costs. To further discuss the impact of compensation levels on outpatient OOP costs for rural residents with different incomes, this section divides the income levels of insured residents into low-income and middle- and high-income groups based on median income and discusses them separately. Table 3 reports the effects of changes in URRBMI compensation levels on outpatient visits and outpatient OOP costs for rural residents. The results in Table 3 indicate that the outpatient visit behavior of residents in the low-income group is more sensitive to the outpatient deductible and outpatient ceiling line than that of the middle- and high-income group, i.e., lowering the threshold of the outpatient deductible and increasing the outpatient ceiling line more significantly increase the willingness of residents in the low-income group to make outpatient visits. The willingness of participants in the middle and high-income groups to receive outpatient visits is not affected by the outpatient ceiling line, but too high an outpatient deductible and too low an outpatient ceiling line will also increase their outpatient OOP costs.

**Table 3:** Effect of level of compensation on outpatient visits and outpatient OOP costs for different income groups of rural residents.

Variables	Low-income group		Upper middle-income group	
	Stage 1: whether outpatient	Stage 2: OOP costs for outpatient visits	Stage 1: whether outpatient	Stage 2: OOP costs for outpatient visits
Outpatient deductible	−0.003^**^	0.299	−0.004^***^	0.450^*^
	(0.002)	(0.385)	(0.001)	(0.228)
Outpatient reimbursement rate	0.386	−0.896	0.631^**^	−0.455
	(0.321)	(3.353)	(0.308)	(1.061)
Outpatient ceiling line	0.025^**^	0.931	0.014	−0.188^**^
	(0.012)	(0.686)	(0.010)	(0.087)
Inverse mills ratio		−4.033		0.011
		(2.589)		(1.475)
Control variables	YES	YES	YES	YES
Constant term	−0.337	8.323^**^	−1.082^**^	7.874
	(0.325)	(3.712)	(0.454)	(4.876)
N	4,627	32	5,114	35

Robust standard errors in parentheses, ^***^, ^**^, and ^*^ are significance levels of 1, 5, and 10%, respectively.

Table 4 reports the impact of the URRBMI compensation levels on rural residents’ inpatient visits and inpatient OOP costs in different income groups. The results in Table 4 indicate that the inpatient OOP costs of rural residents in the low-income group are more likely to be affected by the inpatient reimbursement rate than those in the upper-middle-income group, i.e., an increase in the inpatient reimbursement rate significantly reduces their OOP inpatient costs. The results from Tables 3, 4 collectively demonstrate that rural low-income groups are more sensitive to adjustments in URRBMI compensation levels. This heightened sensitivity can be attributed to the budget constraints faced by low-income populations, which amplify their price elasticity of demand for healthcare services. Measures such as lowering deductibles, raising reimbursement rates, or increasing ceiling lines directly reduce the initial financial barriers to healthcare access, thereby significantly stimulating demand among this demographic. In contrast, higher-income groups exhibit lower elasticity of healthcare demand, prioritizing service quality over cost considerations. Consequently, adjustments to compensation levels exert minimal influence on their healthcare-seeking behavior.

**Table 4:** Effect of level of compensation on inpatient visits and OOP inpatient costs by income group of rural residents.

Variables	Low-income group		Upper middle-income group	
	Stage 1: whether inpatient	Stage 2: OOP costs for inpatient visits	Stage 1: whether inpatient	Stage 2: OOP costs for inpatient visits
Inpatient deductible	0.085	0.326	−0.133^**^	0.390^*^
	(0.060)	(0.237)	(0.062)	(0.233)
Inpatient reimbursement rate	0.238	−3.174^**^	−0.209	0.111
	(0.287)	(1.509)	(0.248)	(1.778)
Inpatient ceiling line	0.032	0.187	0.051	−0.273
	(0.050)	(0.202)	(0.041)	(0.174)
Inverse mills ratio		1.292		−0.128
		(0.817)		(0.633)
Control variables	YES	YES	YES	YES
Constant term	−1.669^***^	6.990^**^	−0.082	11.110^***^
	(0.580)	(3.436)	(0.724)	(2.998)
N	4,818	245	6,661	272

Robust standard errors in parentheses, ^***^, ^**^, and ^*^ are significance levels of 1, 5, and 10%, respectively.

To explore the impact of compensation level on the utilization of healthcare resources and medical Taking into account the sample size, the samples in this section were divided into the low-health group and the upper-middle health group. The low-health group examined the samples whose self-assessed health was very unhealthy and relatively unhealthy, while the upper-middle health group examined the samples whose self-assessed health was very healthy, relatively healthy, and generally healthy. The results obtained are shown in Table 5.

**Table 5:** Effect of level of compensation on outpatient visits and outpatient OOP costs for different health groups of rural residents.

Variables	Low-health group		Upper middle-health group	
	Stage 1: whether outpatient	Stage 2: OOP costs for outpatient visits	Stage 1: whether patient	Stage 2: OOP costs for outpatient visits
Outpatient deductible	−0.004^***^	0.002^***^	−0.001	0.009^**^
	(0.001)	(0.001)	(0.001)	(0.003)
Outpatient reimbursement rate	0.442^*^	−0.708^*^	1.027^***^	−1.25
	(0.239)	(0.412)	(0.277)	(1.004)
Outpatient ceiling line	0.018^**^	−0.010	0.022^**^	0.010
	(0.009)	(0.025)	(0.009)	(0.031)
Inverse mills ratio		0.200		−0.126
		(0.564)		(0.907)
Control variables	YES	YES	YES	YES
Constant term	−0.374	7.380^***^	−1.150^***^	7.578^***^
	(0.248)	(0.984)	(0.316)	(1.729)
N	7,319	684	7,082	372

Robust standard errors in parentheses, ^***^, ^**^, and ^*^ are significance levels of 1, 5, and 10%, respectively.

Table 5 reports the effects of URRBMI compensation levels on outpatient visits and outpatient OOP costs for rural residents in different health groups. The results in Table 5 indicate that for rural residents in the low-health group, lower outpatient thresholds, higher reimbursement rates, and higher ceiling lines can significantly increase outpatient visits and that the willingness of rural residents in the low-health group to attend outpatient visits is more likely to be affected by the thresholds than those in the upper middle-health group. In terms of outpatient OOP costs, lowering the outpatient deductible can significantly reduce the costs of outpatient visits for all rural residents, and increasing the reimbursement rate has a more significant effect on reducing the outpatient OOP costs of rural residents in poorer health.

Overall, lowering the deductible for outpatient visits and raising the reimbursement rate and ceiling line can effectively release the demand for outpatient visits from less healthy residents, and lowering the deductible and raising the reimbursement rate can also effectively reduce the costs of outpatient visits on rural residents.

Table 6 reports the impact of changes in the URRBMI compensation levels on inpatient visits and inpatient OOP costs for residents in different health groups, with the sample similarly differentiated into low and medium-high health groups. The results in Table 6 indicate that the willingness to visit the hospital and inpatient OOP costs are more significantly affected by the inpatient reimbursement rate and inpatient ceiling line for residents in the low-health group than for those in the healthier group, i.e., the increase in the inpatient reimbursement rate and inpatient ceiling line significantly releases the willingness to be hospitalized for residents in the poorer health level. At the same time, the increase in the inpatient ceiling line may also have raised rural residents’ inpatient OOP costs, possibly as a result of a significant increase in willingness to seek medical treatment following the increase in the compensation level.

**Table 6:** Effect of level of compensation on inpatient visits and OOP inpatient costs for different health groups of rural residents.

Variables	Low-health group		Upper middle-health group	
	Stage 1: whether inpatient	Stage 2: OOP costs for inpatient visits	Stage 1: whether inpatient	Stage 2: OOP costs for inpatient visits
Inpatient deductible	−0.017	−0.009	−0.054^*^	−0.04
	(0.028)	(0.156)	(0.030)	(0.150)
Inpatient reimbursement rate	0.325^**^	−2.033^*^	0.174	−0.391
	(0.161)	(1.232)	(0.186)	(2.006)
Inpatient ceiling line	0.087^**^	0.230^*^	0.042	0.398^**^
	(0.038)	(0.132)	(0.045)	(0.179)
Inverse mills ratio		1.863^***^		2.564^**^
		(0.638)		(1.009)
Control variables	YES	YES	YES	YES
Constant term	−1.377^***^	6.287^**^	−1.504^***^	3.406
	(0.483)	(2.491)	(0.573)	(3.942)
N	10,432	407	9,822	111

Robust standard errors in parentheses, ^***^, ^**^, and ^*^ are significance levels of 1, 5, and 10%, respectively.

### 4.3 Robustness check

The Heckman two-stage regression method can solve the self-selection problem of the sample to a certain extent, but there may be the problem of omitted variables. To further address the problem of omitted variables in the model and to test its robustness, this study further explores the double fixed-effects double-difference model, which controls for the effects of omitted variables by introducing time-fixed effects and regional effects. In this part, the ‘with deductible’ and ‘without deductible’ groups, the ‘high reimbursement rate’ group and the ‘low reimbursement rate group’ group were explored separately using the double difference model with fixed effects, “low reimbursement rate group”, “with ceiling line” and “without ceiling line” groups of rural residents in the use of medical resources and medical costs, the results are shown in Supplementary Tables S8–S13.

The results in Supplementary Tables S8–S13 show that (1) after the implementation of the URRBMI integration policy, the probability of outpatient visits for rural residents in areas with a deductible is significantly lower, and outpatient OOP costs are significantly higher, while there is no significant change in outpatient visit behavior and outpatient OOP costs for the sample in the group without a deductible. This suggests that setting an outpatient deductible significantly reduces rural residents’ outpatient medical resource utilization behavior and increases outpatient visit costs, whereas not setting a deductible is meaningful for outpatient visit cost control, and the discussion of the inpatient ceiling line has similar results. (2) Increasing the reimbursement rate for outpatient visits did not significantly release the demand for outpatient visits among rural residents and increasing the reimbursement rate for inpatient can significantly increase the willingness of rural residents to receive inpatient visits, but it may also raise certain moral hazard problems. (3) URRBMI integration can significantly increase the willingness of rural residents in the high ceiling line group to visit the hospital, i.e., the higher the ceiling line is, the higher the willingness of rural residents to make inpatient visits; at the same time, the treatment model without outpatient ceiling line is more favorable to alleviate the burden of residents’ outpatient visits.

In conclusion, after solving the endogeneity problem, the URRBMI integration policy to increase the compensation level is meaningful in alleviating the medical costs of rural residents, similar to the previous results, and passes the robustness test.

## 5 Discussion

### 5.1 Similarities and differences with existing studies

Similar to existing studies, the results of this study validate the role of health insurance reimbursement levels, including deductible, insurance percentage, and ceiling line, in medical costs (20–27).

Unlike previous related studies on URRBMI integration, most of the previous studies focused only on the implementation effects of health insurance integration or not, including the discussion of healthcare resource utilization, medical costs, health effects, and equity issues, while this study is innovative in its research perspective by adding compensation schemes to the discussion of URRBMI integration on OOP medical costs of rural residents. At the same time, this study combed the data on the level of URRBMI coverage in 125 cities in China, which can comprehensively represent the process of URRBMI integration in China, and is of positive significance to the discussion of China’s healthcare reform.

### 5.2 Limitations and future recommendations

Although this study has some significance for improving health care reform for rural residents in China and other developing countries, there are some shortcomings in this study:

First, at the data time level, this study uses data from the CHARLS database, which is currently updated only to 2020. The implementation of the URRBMI integration policy is a long-term process, and due to data limitations, this study does not explore the long-term policy effects of this policy. The impact of the implementation of URRBMI integration on rural residents may also have a lag.

Second, at the level of the research object, this study uses CHARLS data to include only the group over 45 years old, and the welfare protection of rural children, adolescents, and young people is also an important issue that needs to be explored. The above issues can be further improved in future studies.

## 6 Conclusion and policy implications

### 6.1 Conclusion

Rural social security is a priority issue for reform in developing countries such as China. The central question of this study is whether the compensation level of URRBMI integration has alleviated the OOP medical costs of rural residents. In this regard, this chapter uses a sample of residents in areas where URRBMI integration has been implemented and employs Heckman’s two-stage regression to analyze the impact of the compensation level of urban–rural residents’ medical insurance integration on rural residents’ healthcare behaviors and OOP medical costs, as well as differentiating between the residents’ income level and their health level to conduct heterogeneity analyses. To further control the problem of omitted variables, a double difference model controlling for time effects and area effects was used to test for endogeneity and robustness issues. The main findings of the study are as follows:

First, the compensation level of URRBMI integration in China has been rising at a slower pace. From 2015 to 2020, in the outpatient compensation level of China’s URRBMI, the outpatient deductible has gradually declined and the outpatient reimbursement rate has tended to rise, while the outpatient ceiling line has not changed much or even tended to decline. In the inpatient compensation level, the adjustment of the deductible in tertiary care institutions is obvious, while the increase in the inpatient reimbursement rate and inpatient ceiling line is slower.

Second, raising the compensation level has, to a certain extent, released rural residents’ demand for outpatient care and eased OOP medical costs. Rural residents’ demand elasticity for outpatient visits is greater than that for inpatient visits. Reducing the deductible for outpatient visits and raising the reimbursement rate and ceiling line for outpatient visits will significantly release residents’ demand for outpatient visits, and the key to easing residents’ outpatient OOP costs is to lower the deductible and ceiling line. At the same time, the current URRBMI integration of the compensation level increase has not been effective in raising the willingness of rural residents to inpatient visits, rural residents’ inpatient costs for inpatient reimbursement rate are more sensitive.

Third, raising the compensation level is conducive to releasing the demand for medical care from vulnerable groups, including rural low-income groups and groups with poorer health, and is also meaningful in easing the burden of medical care. Lowering the deductible, increasing the reimbursement rate, and raising the ceiling line is effective in increasing outpatient OOP visits and inpatient visits for disadvantaged groups, and the key to easing outpatient OOP costs for rural low-income groups is to raise the reimbursement rate and the ceiling line. The consultation behavior of rural middle and high-income groups is less affected by the compensation level. Effective identification of vulnerable groups is imperative in the setting of compensation levels for URRBMI integration.

### 6.2 Policy recommendations

The results of this study have important policy implications for the optimization of health insurance systems in developing countries such as China. On the one hand, it is necessary to consider the role of compensation-level settings in assessing the effectiveness of health insurance policies. On the other hand, the differentiated treatment content of health insurance can lead to different outcomes, and rural low-income groups and groups with a high burden of disease are still the key targets of medical insurance. Although the URRBMI integration policy has improved many aspects, the current effect of raising the compensation level on reducing residents’ OOP medical expenses is not strong. Thus, in terms of policy formulation for the reimbursement rate of the URRBMI treatment level, there is still a need to further reduce the contribution burden of low-income groups, lower the deductible line for visits by low-income groups, and increase the reimbursement rate for visits by residents with serious illnesses as well as the ceiling line limit. It is also necessary to expand the catalog of medicines and treatment items reimbursed under the URRBMI, to reduce the limitations imposed by economic constraints on the medical needs of the population on a wider scale, and to provide more financial support to families that are impoverished due to illness. Specifically, first of all, we need to improve the hierarchical diagnosis and treatment, and reasonably set the compensation level, due to the heterogeneity of the individual economic status of the residents, the health insurance coordination of the medical burden of different income groups has different impacts. In the future, it is necessary to set up a mechanism to link the content of medical insurance treatment with personal income. Second, optimize the financing scheme and improve the financing mechanism. China currently adopts a financing method based on a fixed amount of contribution, which is still lacking in terms of financing equity. In the future, in terms of the design of the financing level, we can learn from the financing programs of medical insurance in Japan, Germany, the United States, and other countries, and use income as the criterion for raising the medical insurance fund, without considering other factors. The design of the level of protection can also be modeled on Japan’s practice of differentiating the level of protection according to income levels, with participants of higher economic levels bearing a higher deductible threshold. Finally, the purchase of commercial insurance should be encouraged in a targeted manner to meet the needs of different groups.

This study systematically discusses the overall impact of the compensation level of the URRBMI integration policy on residents’ medical costs, and there is still room for future exploration in the following research directions: (1) Explore the long-term impact of the URRBMI policy on rural residents’ medical costs. (2) Using hospital reimbursement data to discuss in more detail the impact of outpatient visits and inpatient hospitalization on the deductible, reimbursement rate, and ceiling line on patients’ behavior and medical costs. (3) Explore specific solutions to optimize the level of medical insurance coverage, such as calculating the optimal compensation level based on the objectives of efficiency and equity, respectively. (4) Explore targeted health insurance protection mechanisms for vulnerable groups in rural areas.

## Data Availability

The data analyzed in this study is subject to the following licenses/restrictions: the data on thresholds, reimbursement rates and ceiling lines involved in this study were compiled by the author’s team, please contact the authors if you need to obtain them. Requests to access these datasets should be directed to Chen Liu, 805878438@qq.com.
